# Higher livestock abortion burden in arid and semi-arid lands, Kenya, 2019–2020

**DOI:** 10.1371/journal.pone.0297274

**Published:** 2024-02-22

**Authors:** John Gachohi, Peris Njoki, Eddy Mogoa, Fredrick Otieno, Mathew Muturi, Athman Mwatondo, Isaac Ngere, Jeanette Dawa, Carolyne Nasimiyu, Eric Osoro, Bernard Bett, Kariuki Njenga

**Affiliations:** 1 Department of Environmental Health and Disease Control, School of Public Health, Jomo Kenyatta University of Agriculture and Technology, Nairobi, Kenya; 2 Washington State University Global Health Program, Washington State University, Nairobi, Kenya; 3 Paul G. Allen School of Global Health, Washington State University, Pullman, Washington, United States of America; 4 Department of Clinical Studies, Faculty of Veterinary Medicine, University of Nairobi, Nairobi, Kenya; 5 Animal and human health Program, International Livestock Research Institute, Nairobi, Kenya; 6 Kenya Zoonotic Disease Unit, Nairobi, Kenya; 7 Dahlem Research School (DRS), Faculty of Veterinary Medicine, Freie Universität Berlin, Berlin, Germany; 8 Kenya One Health Platform, Ministry of Health, Nairobi, Kenya; National Veterinary Research Institute (NVRI), NIGERIA

## Abstract

Tracking livestock abortion patterns over time and across factors such as species and agroecological zones (AEZs) could inform policies to mitigate disease emergence, zoonoses risk, and reproductive losses. We conducted a year-long population-based active surveillance of livestock abortion between 2019 and 2020, in administrative areas covering 52% of Kenya’s landmass and home to 50% of Kenya’s livestock. Surveillance sites were randomly selected to represent all AEZs in the country. Local animal health practitioners electronically transmitted weekly abortion reports from each ward, the smallest administrative unit, to a central server, using a simple short messaging service (SMS). Data were analyzed descriptively by administrative unit, species, and AEZ to reveal spatiotemporal patterns and relationships with rainfall and temperature. Of 23,766 abortions reported in all livestock species, sheep and goats contributed 77%, with goats alone contributing 53%. Seventy-seven per cent (n = 18,280) of these abortions occurred in arid and semi-arid lands (ASALs) that primarily practice pastoralism production systems. While spatiotemporal clustering of cases was observed in May-July 2019 in the ASALs, there was a substantial seasonal fluctuation across AEZs. Kenya experiences high livestock abortion rates, most of which go unreported. We recommend further research to document the national true burden of abortions. In ASALs, studies linking pathogen, climate, and environmental surveillance are needed to assign livestock abortions to infectious or non-infectious aetiologies and conducting human acute febrile illnesses surveillance to detect any links with the abortions.

## Introduction

Livestock production is central to Kenya’s economy, contributing 4.4% of the gross domestic product (GDP) (USD 3.4 billion, in 2017) [1]. Small-scale livestock production is primarily practiced in rural areas, accounting for 90% and 80% of national beef and milk production, respectively [1]. By keeping one or more livestock of cattle, sheep, goat, camel, or donkey species, resource-poor households access animal-source foods while enhancing other livelihood aspects such as healthcare and education [2]. Livestock abortions remain a significant constraint to satisfactory reproductive performance in sub-Saharan Africa (SSA), causing direct and indirect demographic impacts through reduced availability of replacement animals, longer calving intervals, premature culling, and economic impacts through decreased milk production and increased veterinary costs [3–5].

Infectious agents, toxins, trauma, heat stress, and nutritional deficiencies may cause livestock abortions. Many infectious agents that cause livestock abortion are zoonotic with a high risk of transmission to humans, such as *Brucella* spp., Rift Valley fever (RVF) virus, *Coxiella burnetii*, *Leptospira* spp., and *Toxoplasma gondii* among others [6–10]. Non-zoonotic infectious abortifacient agents such as Peste des petits ruminants and lumpy skin disease viruses are associated with high mortality and production losses in livestock [11, 12].

Efficient and reliable surveillance systems are vital for decision-making regarding animal health trends [13]. In response to this, we previously undertook two surveillance studies that demonstrated gaps and burden of livestock abortions—an active RVF surveillance effort where we found that livestock abortions accounted for 37% of the reported RVF-associated syndromes [14]. Our other study that used an open-source mobile phone-based disease reporting system among domestic and wild animals revealed a high frequency of livestock abortions exceeded only by respiratory, gastrointestinal and skin diseases [15]. Notably, these and other existing data on livestock abortion in Kenya has focused on specific diseases, locations, or species. Consequently, they have not provided a comprehensive overview that can be tracked and compared over time. Nevertheless, structured and robust surveillance of livestock abortions could inform prevention and control approaches that enhance livestock production and serve as an early warning of many zoonoses, including emerging and re-emerging diseases. Here, we conducted a 12-month active surveillance to determine the burden of livestock abortion in Kenya using a large longitudinal cohort in 523 randomly selected small administrative units (wards) covering 52% of Kenya’s landmass and home to 50% of Kenya’s livestock population.

## Methods

### Selection of study sites

As per the 2010 constitution, Kenya is administratively divided into 47 semi-autonomous counties which are further divided into 290 sub-counties (districts) and finally, wards. Agriculturally, the country is divided into seven agroecological zones (AEZs) based on soil types, landforms, and climatic conditions resulting in varied agricultural potential. These include the agro-alpine/humid, high potential, medium potential, semi-arid, arid, very arid and desert land characterized by high to low agricultural potential in that order. For the purpose of sampling in this study, we collapsed these seven zones into five zones that included (i) agro-alpine/humid, (ii) high and medium potential, (iii) semi-arid, (iv) arid, and (v) very arid/desert. Most of the livestock population, 52%, reside in the arid and very arid zones, followed by 14% in the semi-arid zone. The high and medium potential zones have 8% and 15% of the animals, respectively. The rest (11%) reside in the agro-alpine/humid zone.

**Fig 1:**
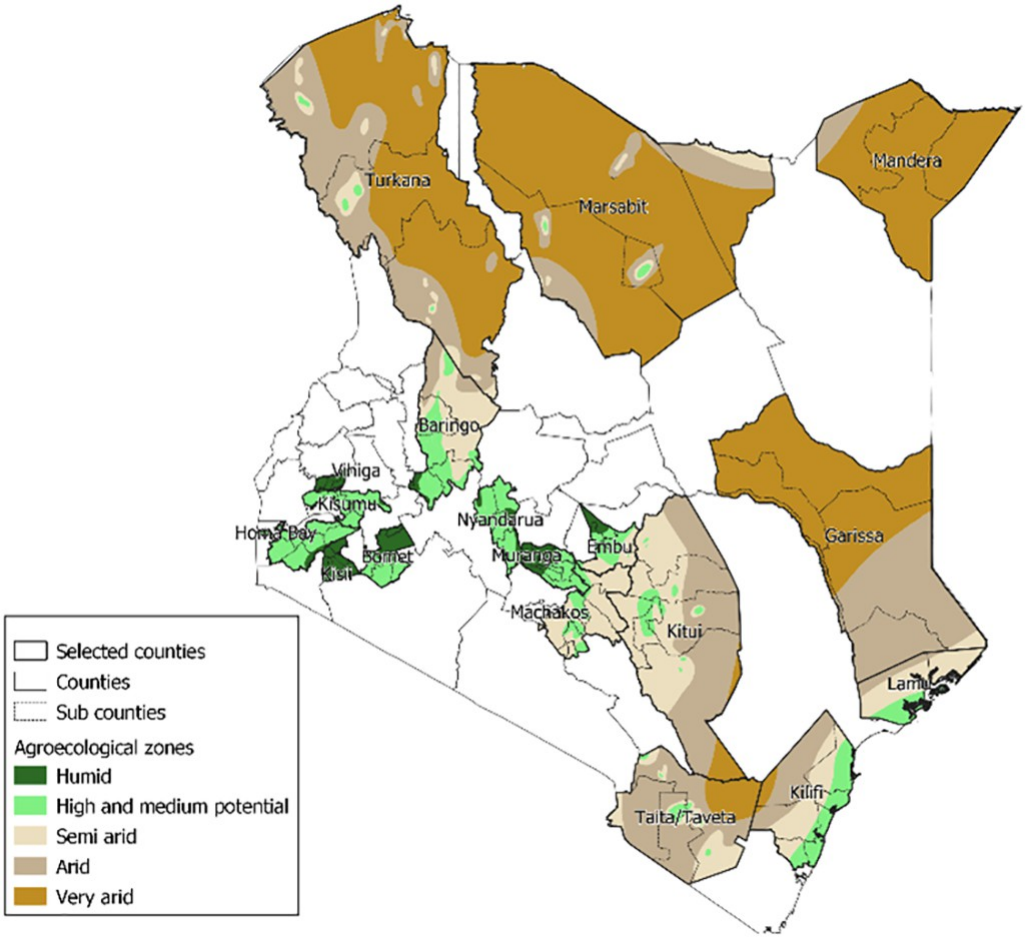
Distribution of the 18 counties (in which the 523 wards lie) selected by agroecological zone.

We aligned each of the 290 sub-counties in the country to the dominant AEZ and selected four sub-counties randomly in each of the five collapsed AEZs. We then identified the counties where each of these 20 sub-counties fell. To increase the geographical spread of the study, we included the rest of the sub-counties in each of identified counties. We had anticipated ending up with 20 counties, but in the process of random sampling, we ended up with 18. Fig 1 shows the distribution of the 18 counties (in which the 115 study sub-counties lie) selected by AEZ. We then implemented active livestock abortion surveillance in the 18 counties to determine the burden of abortion among livestock in Kenya. Five hundred and twenty three wards are nested within 115 sub-counties. We recruited ward-level animal health practitioners (AHPs) for data collection in January to April 2019.

### Data collection and analyses

This study was implemented over 52 weeks between April 2019 to June 2020 across 18 counties. Abortion events in all livestock species, including cattle, sheep, goat, camel, and donkey, were recorded at the ward level. Weekly abortion reports from the AHPs were electronically transmitted to Zoonotic Disease Unit (ZDU). The ZDU is a collaboration between the animal and human health ministries in the Government of Kenya, whose creation is part of realizing the One Health approach in Kenya to manage zoonoses. The AHPs used a data collection tool that gathered information about the number and species involved. Data collection in the field by the ward AHPs engaged a simple short messaging service (SMS) prompted by the server every Friday. The users interacted with the platform by answering questions via the standard SMS service. Each interaction defined how platform users moved through the flow of their responses. For instance, "*Did your ward experience livestock abortions in the last week*?" Responses "yes" or "no" were recorded and sent. If a "yes" response was sent, another message prompted the AHP to record the number and the species involved. The texts were sent directly to the AHPs’ cell phones, and response data were stored on the online secure, encrypted platform. The text-gathered data were downloaded into Ms Excel for analysis. We analyzed the time-series variations of livestock abortions across time, counties, species and AEZ to reveal any abnormal outcome variation. We used Quantum Global Information System (QGIS) version 3.4.4 software (https://qgis.org) and R software version 3.6.2 (https://cran.r-project.org/) to generate choropleth maps that revealed the distribution of abortions by time, counties, species and AEZ. The study sourced shapefiles from GADM that provides maps and spatial data for all countries and their sub-divisions (https://gadm.org/). We used the Kenya livestock census data of 2009 to provide denominators for species comparisons where necessary. To reveal spatiotemporal patterns and relationships with rainfall and temperature, we extracted rainfall from the Climate Hazards Group InfraRed Precipitation with Station (CHIRPS) dataset [16]. CHIRPS data are superior and reliable compared to other satellite-based precipitation products especially for data scarce locations such as hard-to-reach ASAL areas [16]. The association between abortions and rainfall and temperature was assessed using the Pearson´s correlation coefficients.

## Results

### Characteristics of the study area

We conducted this study in 18 (38%) of 47 counties in Kenya, covering 52% of the country’s landmass. These 18 counties are home to 37,618,692 livestock (cattle, sheep, goats, camels, donkeys) and 523 wards [17]. The predominant livestock is goats (42%), sheep (28%), and cattle (23%), whereas the camel (6.5%) and pig (0.5%) populations are the lowest.

### Epidemiological characteristics

**Fig 2:**
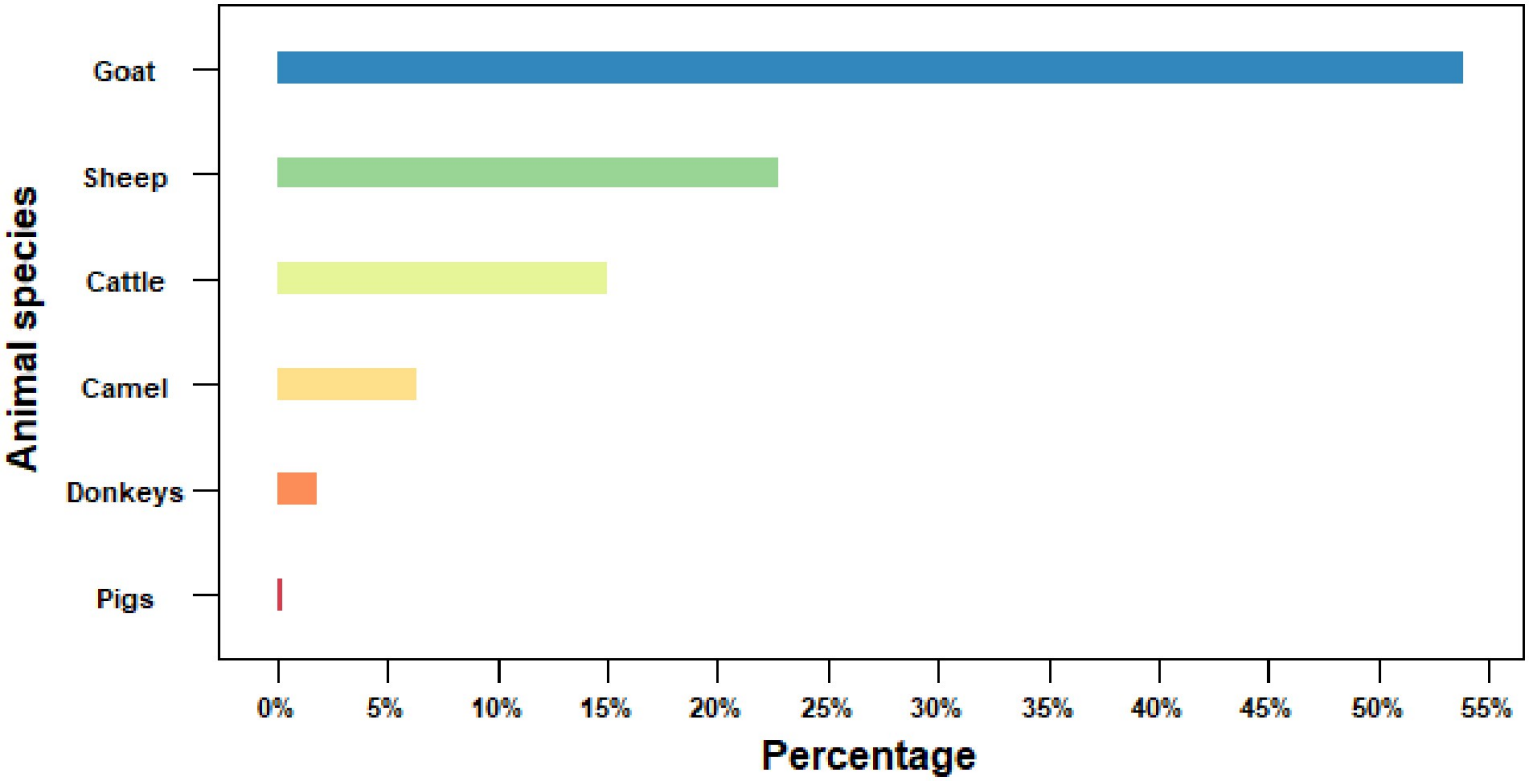
Proportion of livestock abortions (%) across 18 counties of Kenya, 2019.

Between April 2019 and June 2020, 23,766 livestock abortions were reported in the 18 study counties, with sheep and goats contributing 77% of the abortions. Goats contributed 53% of the cases, while only 15% of the abortions were reported in cattle (Fig 2).

### Burden of abortions among livestock across counties

**Fig 3:**
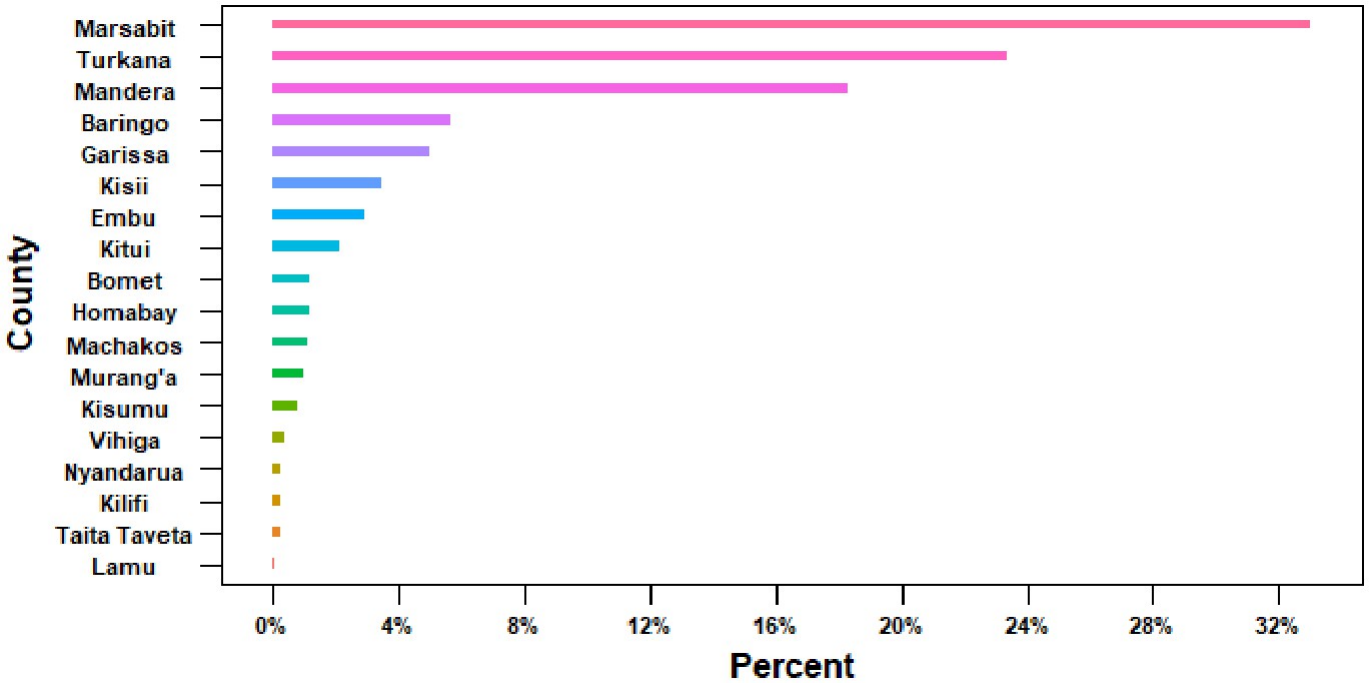
Proportion of abortion events (%) by county across all livestock species in Kenya 2019.

**Fig 4:**
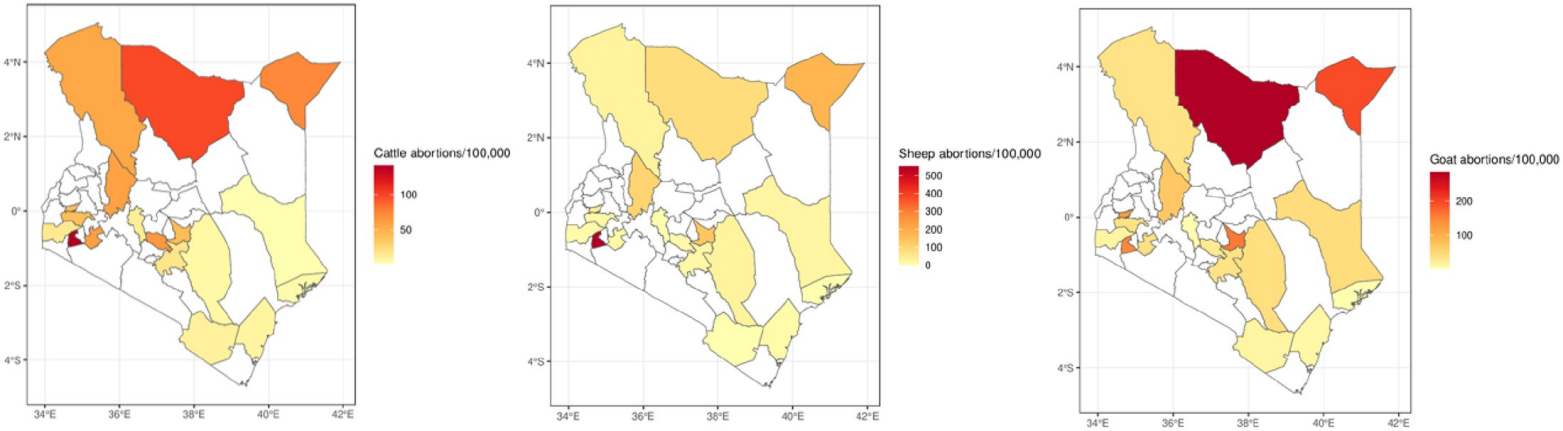
Abortions per 100,000 cattle (left), sheep (middle) and goats (right) across counties in Kenya 2019–2020.

Two-thirds of all livestock abortions were reported from two neighbouring counties, Marsabit and Turkana, located in northern Kenya’s ASALs (Figs 3 and 4). Similarly, three other counties in the ASALs (Mandera, Baringo and Garissa–refer to Fig 1) contributed 19% of all abortion reports (Fig 3).

### Incidence of abortions across livestock species

Fig 4 shows choropleth maps of abortion reports per 100,000 animals by species. High burdens were revealed in all counties in the ASAL regions and three counties with agro-alpine/humid and high and medium AEZs, i.e., Embu, Kisii and Vihiga (Fig 1 and Table 1).

**Table 1:** Annual abortion rates per 100,000 by species and predominant AEZs.

County	Cattle	Goats	Sheep	Predominant AEZs
Baringo	59	69	103	Arid and semi-arid
Garissa	3	43	24	Arid and semi-arid
Kilifi	9	11	11	Arid and semi-arid
Kitui	8	41	30	Arid and semi-arid
Mandera	74	194	176	Arid and semi-arid
Marsabit	98	287	81	Arid and semi-arid
Turkana	56	36	30	Arid and semi-arid
Bomet	61	38	17	High and medium potential
Homa Bay	16	13	10	High and medium potential
Kisumu	41	35	18	High and medium potential
Machakos	18	35	19	High and medium potential
Nyandarua	13	1	3	High and medium potential
Taita Taveta	11	14	0	Medium potential, arid and semi-arid
Lamu	6	2	0	Medium potential, arid and semi-arid
Embu	43	160	133	Agro-alpine/humid, high and medium potential
Kisii	141	147	552	Agro-alpine/humid, high and medium potential
Muranga	65	26	11	Agro-alpine/humid, high and medium potential
Vihiga	40	123	23	Agro-alpine/humid, high and medium potential

### Burden of abortions across time

**Fig 5:**
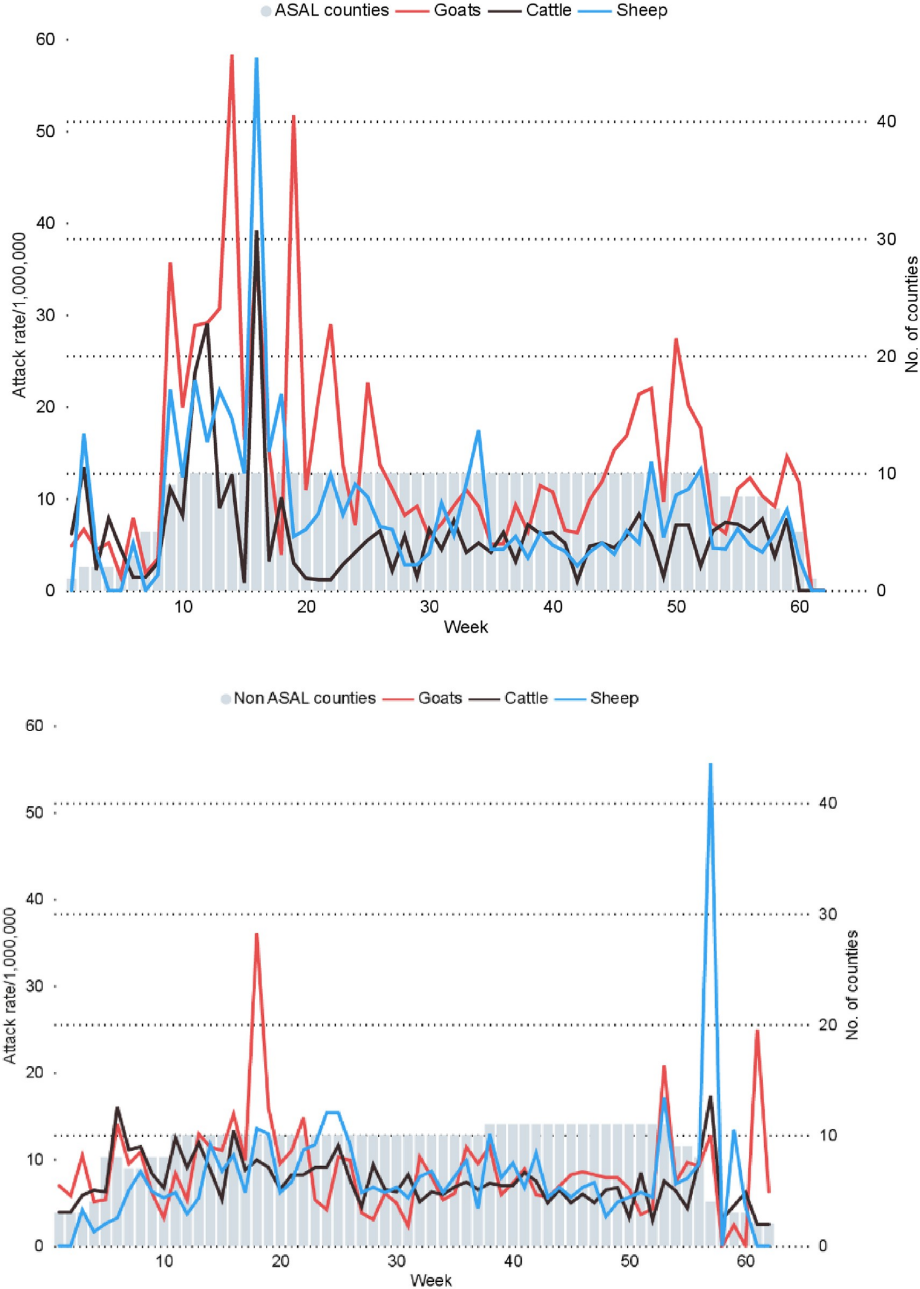
Weekly abortion events per 1,000,000 in cattle, sheep and goats reared in the ASAL zones (top panel) and in the non-ASAL zones (agro-alpine/humid and high and medium potential AEZs) (bottom panel), in Kenya 2019–2020.

Fig 5 shows the temporal evolution of abortion reports collapsed into ASAL and non-ASAL counties. There was an increase in reporting across ASAL counties between May and July 2019, which stabilized with time. Goat abortions were consistently reported across time though they highly overlapped with sheep abortions in the ASALs (Fig 5, top panel). A spike in sheep abortion in non-ASAL counties towards the end of the study was notable (Fig 5, bottom panel).

### Association between abortions, rainfall and temperature

Overall, there was no significant fluctuation in abortions associated with temperature and rainfall changes. However, in the agro-alpine/humid zone, there was a significant negative correlation with rainfall among cattle and goats (Table 2).

**Table 2:** Correlation between abortions and rainfall and temperature within AEZs.

AEZ	Species	Climate	Correlation coefficient (95% CI)
Arid and semi-arid	Cattle	Temperature	-0.11 (-0.35, 0.15)
		Rainfall	-0.16 (-0.40, 0.09)
	Sheep	Temperature	-0.11 (-0.35, 0.14)
		Rainfall	-0.15 (-0.39, 0.10)
	Goat	Temperature	-0.08 (-0.32, 0.18)
		Rainfall	-0.07 (-0.31, 0.19)
High and medium potential	Cattle	Temperature	-0.19 (-0.42, 0.06)
		Rainfall	0.003 (-0.25, 0.25)
	Sheep	Temperature	0.14 (-0.12, 0.37)
		Rainfall	0.18 (-0.08, 0.41)
	Goat	Temperature	-0.01 (-0.26, 0.24)
		Rainfall	-0.08 (-0.32, 0.16)
Agro-alpine/humid	Cattle	Temperature	-0.02 (-0.27, 0.23)
		Rainfall	-0.26 (-0.47, -0.006)
	Sheep	Temperature	0.21 (-0.04, 0.44)
		Rainfall	-0.19 (-0.42, 0.06)
	Goat	Temperature	-0.05 (-0.30, 0.20)
		Rainfall	-0.32 (-0.53, -0.08)

## Discussion

Using a novel data collection approach that automated SMS-led conversational interactions with AHPs working at the smallest administrative unit, we report population-level estimates of livestock abortion across Kenya. Over one year, 23,766 livestock abortions were reported in 18 study counties covering 52% of the land mass and representing all AEZs. Goats contributed 53% of the abortion cases, while sheep and cattle contributed 24% and 15%, respectively. To our knowledge, these findings are the first to reflect the burden of livestock abortion at the population level in Kenya. Our study also identified the ASALs zone and the May-July season as the most burdened by abortions. While similar studies with comprehensive coverage such as ours are rare in the literature, a prospective abortion cohort study conducted from October 2017 through September 2019 in northern Tanzania determined zoonotic aetiologies of livestock abortions [4]. In neighboring Ethiopia, a study examining small ruminant abortions across three agro-ecologies reported abortions in 59% of goat flocks and 18% of sheep flocks over one year, with a mean annual flock abortion percentage being 16% for goats and 13% for sheep [3]. Our study findings contribute to knowledge on factors impacting animal source foods, reproductive losses, and disease interventions. In addition, our findings open new research avenues to further explore pathogen, climate, environmental and human surveillance to assign abortions to infectious or non-infectious aetiologies and detect pathogen spillover to humans in sub-Saharan Africa.

More than three in every four (77%) of these abortions occurred in arid and semi-arid lands (ASALs) that primarily practice pastoralism production systems. This higher abortion burden in ASALs, often missed by routine surveillance systems, adds to the knowledge that these regions are perhaps more prone to zoonotic infections under high animal-to-human ratios (domestic and wild animals) relative to other AEZs [18–20]. ASALs are characterized by animal movements responding to spatial and temporal heterogeneity in pasture and water availability. These movements generate patterns of contact within populations that have important implications for the spread of pathogens. Therefore, the high abortion burden in ASALs likely reflected the numerous underlying challenges among livestock in these areas [18, 21, 22]. Given the role that livestock abortion plays in the transmission of zoonoses, identifying the predominant reservoir species of zoonotic pathogens and heightening surveillance sensitivity in ASAL zones are necessary steps toward minimizing zoonoses risk.

The lower abortion burden in agro-alpine/humid, high, and medium potential zones likely result from the intensification of livestock systems to stall feeding in response to the increased human population occupying tiny land units in these zones. Intensification is characterized by minimal or no inter-herd contacts and improved disease management systems through biosecurity, natural isolation, vaccination, and other technological options [23]. Studies, for instance, have found lower exposure to abortion-causing brucellosis in intensified systems relative to the ASAL zones [21]. However, other abortion-causing diseases, such as the vector borne-RVF, would still be transmitted in all production systems [24]. So, when we analyzed abortions per 100,000 animals, we revealed high burdens in three non-ASAL counties, i.e., Embu, Kisii and Vihiga (refer to Fig 1), most likely due to the low denominators in those counties or the factors expounded above in intensified production systems. Therefore, we hypothesize that the aetiologies reported in the Tanzanian study may also be responsible for abortions in these areas [4]. This is especially *Neospora caninum*, a known major cause of reproductive failure in cattle herds practicing stall-feeding production systems around the world [25].

We observed prominent differences between species, with goats carrying the heaviest burden per 100,000 animals similar to the study in Ethiopia [3]. This pattern could emanate from intrinsic host traits associated with lifetime reproductive output, i.e., goats have a biannual reproductive cycle representing more opportunities for abortion. However, sheep ranked second after goats, which also reproduce biannually though to a lesser extent [26]. In addition, goats are hardier; they move more frequently in ASALs on short-distance trade interactions and long-distance seasonal interactions, resulting in more complex contact patterns [27], a well-established risk factor for pathogen spread in empirical studies and in prediction models [28, 29]. While the scope of our study excluded data on movements, empirical data covering contact networks of migratory herds is emerging, presenting opportunities for using goats and perhaps sheep as a study system to explore how movement patterns impact abortion of either infectious or non-infectious aetiologies [28, 29].

We sought to explore the influence of rainfall and temperature on the variability of abortions across AEZs and time. Contrary to our expectations, we did not detect a significant corresponding fluctuation in temperature or rainfall with changes in abortions across AEZs. Nevertheless, our data described a spatiotemporal cluster of abortion cases across all species during the May-July 2019 season in ASALs, reflecting a possible outbreak. This was intriguing, suggesting a similar aetiology in the same zones across the country, probably as a result of high temperatures and drought conditions during the study period in ASALs. However, this cluster is unlikely to have resulted from weather changes alone. For instance, in the last ten years, the RVF outbreak phenotype has been changing from the explosive RVF abortion storms experienced between the 1930s-2008 to localized epidemics often missed by surveillance systems [9, 30, 31]. However, there were no reports of human RVF infections in the ASALs during our study period to prove RVF outbreaks. This notwithstanding, the prospective cohort study conducted in northern Tanzania, whose study time partly overlapped with ours and done in an ASAL ecosystem, detected an RVF outbreak in cattle in addition to a high prevalence of *Coxiella burnetii* infection and the first report of *N*. *caninum*, *Toxoplasma gondii*, and pestiviruses [4]. These infections could similarly be causing these abortions in Kenya without being detected. Other non-zoonotic diseases causing abortion outbreaks in sheep and goats in ASAL include Peste des petits ruminants (PPR) but we didn’t find accompanying systemic symptoms, such as massive death, and diarrhoea [11].

While the standard field disease surveillance report from the Notifiable Disease-1 (ND-1) form in Kenya does not provide syndromic data, our abortion burden estimates correlated with data collected using an open-source animal disease reporting system that used mobile phones in collecting data for over two years in other parts of the country [15]. This study, therefore, showcased the underestimation surrounding the ’true’ incidence of abortion rates estimated using the manual surveillance systems in the country. Specifically, surveillance systems fail to capture livestock abortion events at two distinct levels: from the animal herds and flocks due to limited access to veterinary authorities in ASALs, and the failure to report the syndrome via ND-1 form at the national level. Therefore, the biggest strength of this study lies in the use of active surveillance and the short messaging approach that did not require smartphones, which overcame these two surveillance barriers to capture the near-accurate burden of livestock abortion in representative AEZs in the country.

Our study findings add to the knowledge we have gained working on infectious diseases in the ASALs over time. However, surveillance in these areas has been hampered by a lack of proper diagnostics leading to a poor definition of pathogen diversity. In a sense, this leads to suboptimal or incorrect national reporting to the global health security systems. Our findings, therefore, make a good case for expanding surveillance for unknown or emerging pathogens, prioritizing ASALs, and linking it with climate, environmental and human acute febrile illnesses. In ASAL settings, we call for more studies focusing on isolating pathogens from livestock abortion sources and frequent active surveillance for abortion outbreaks [4]. Monitoring for unexplained abortion outbreaks targeting human pathogens might discover emerging pathogens during abortion outbreaks such as those experienced in the May-July 2019 season in this study. Indeed, unknown pathogens could proliferate under favourable climatic factors. Recently, scientists characterized a pathogen landscape using an open-source cloud-based bioinformatics tool in low-resource settings in Cambodia using metagenomic next-generation sequencing in sera. This technology can quickly be adopted using archived and routinely collected sera in ASALs [32].

Nevertheless, our study had limitations. We had only a year to collect the crude data and our results could be biased by the short study time, including failure to adjust for different pregnancy durations and conceptions across species. Data to support that kind of analysis would be logistically unfeasible to collect. However, our data support the case for greater attention to goats in ASALs, which our study has flagged as both animal reproductive health and public health issue.

## Conclusion

To our knowledge, this study represents the most extensive active surveillance on livestock abortion conducted in LMICs revealing the high abortion burdens. First, the study contributes to active surveillance approaches that apply novel text messaging that do not require smartphones. Second, the study revealed a high livestock abortion burden among goats, especially in ASALs in Kenya. Reducing abortion rates is therefore needed to contain zoonoses risk and reproductive wastage. Third, our findings offer valuable information to prioritize public health policies against abortifacient agents, many of which are zoonotic. Finally, our data open new research avenues to further explore pathogen, climate, environmental and human surveillance to assign abortions to infectious or non-infectious aetiologies accurately and detect any pathogen spillover to humans.
